# Supplementary material for: Optimization of Eugenol, Camphor, and Terpineol Mixture Using Simplex-Centroid Design for Targeted Inhibition of Key Antidiabetic Enzymes
Source: Curr Issues Mol Biol. 2025 Jul 2;47(7):512. doi: 10.3390/cimb47070512 (PMC12293521; doi:10.3390/cimb47070512)
Supplement: Supplementary file 1 [file cimb-47-00512-s001.zip › cimb-3729933-supplementary.pdf]

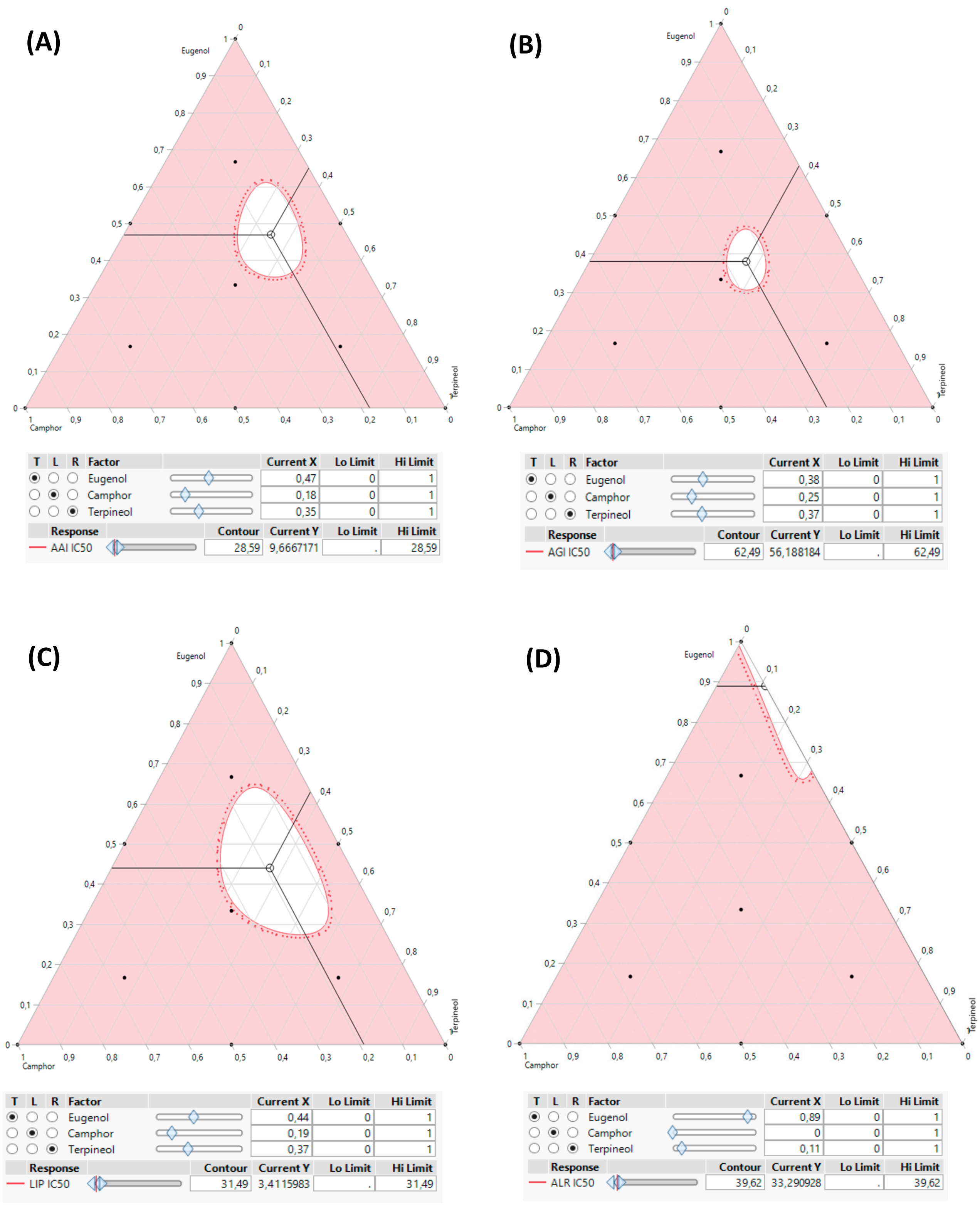

Figure S1. The optimal inhibition values were determined through an in-depth analysis of 2D mixture plots, focusing on the identified compromise zone. Panels (**A**, **B**, **C**, and **D**) display mixture plots that highlight the desired compromise region, located within the ternary mixing zone between the molecules. This zone represents the optimal conditions for achieving maximum AAI, AGI, LIP, and ALR activities, respectively.
